# Nomogram for predicting postoperative recurrence in patients with microvascular invasion-negative hepatocellular carcinoma: development and validation

**DOI:** 10.3389/fimmu.2025.1614392

**Published:** 2025-10-07

**Authors:** Qingwang Ye, Yi Yu, Shujie Pang, Dongbo Zhao, Dongqian Li, Yao Ma, Ning Yang, Wei Feng

**Affiliations:** ^1^ Department of Hepatobiliary Surgery, The Affiliated Suqian Hospital of Xuzhou Medical University, Suqian, China; ^2^ Department of Gastroenterology, The Affiliated Suqian Hospital of Xuzhou Medical University, Suqian, China; ^3^ Department of Hepatobiliary Surgery, Eastern Hepatobiliary Surgery Hospital, Second Military Medical University, Shanghai, China

**Keywords:** hepatocellular carcinoma, microvascular invasion negative, hepatectomy, recurrence, AFP, nomogram, Ki-67

## Abstract

**Background:**

Hepatocellular carcinoma (HCC) imposes a substantial global health burden, while postoperative recurrence remains a pivotal factor contributing to poor prognosis. Although existing prognostic models predominantly focus on patients with HCC with microvascular invasion (MVI), recurrence mechanisms and risk stratification in those with MVI-negative HCC remain underexplored despite their distinct clinicopathological profiles. As such, this study aimed to develop a prognostic nomogram to predict recurrence-free survival (RFS) in patients with MVI-negative HCC.

**Methods:**

Data from 547 treatment-naïve patients with MVI-negative HCC were divided into 2 cohorts: training (n=375); and external validation (n=172). Random survival forest and multivariate Cox regression analyses were used to identify independent prognostic factors. A nomogram prediction model was developed based on risk factors identified in the training cohort and subsequently validated in the external validation cohort.

**Results:**

Key findings revealed that Ki-67, alpha-fetoprotein (AFP)-L3, neutrophil-to-lymphocyte ratio, AFP, and systemic immune-inflammation index significantly impacted RFS, with a concordance-index (C-index) exceeding 0.7 for the nomogram model in the training cohort, and an area under the receiver operating characteristic curve (AUC) of 0.758, 0.769, and 0.779 for 1-, 3-, and 5-year RFS, respectively. The external validation cohort corroborated these findings, achieving C-index values > 0.7 and AUC values of 0.717, 0.735, and 0.756 for the same time points. The calibration curves indicated strong agreement between the predicted and actual outcomes. Decision curve analysis revealed that the nomogram model demonstrated good net benefits for 1-, 3-, and 5-year RFS in both the training and external validation cohorts.

**Conclusion:**

This study developed and validated a prognostic nomogram for predicting postoperative disease recurrence in patients with MVI-negative HCC, highlighting the importance of individualized patient management based on the risk factors identified.

## Introduction

Hepatocellular carcinoma (HCC) poses a formidable global health challenge, with > 860,000 new cases diagnosed annually worldwide (1, 2). China bears a disproportionate burden, accounting for nearly 50% of global HCC incidence and mortality, driven predominantly by endemic hepatitis B virus infection, dietary aflatoxin exposure, and metabolic dysfunction-associated steatotic liver disease (i.e., “MASLD”) (3). Although curative surgical resection provides therapeutic opportunities for early stage HCC, 50%–70% of patients experience recurrence within 5 years, resulting in persistently dismal long-term survival rates (4). This recurrence-driven mortality underscores the critical need for a refined prognostic stratification strategy to optimize postoperative surveillance and adjuvant therapies.

Microvascular invasion (MVI), identified in 20%–50% of resected HCC specimens, is a well-established independent predictor of early disease recurrence and poor prognosis (5–7). Contemporary prognostic models predominantly incorporate MVI status along with tumor size, serum alpha-fetoprotein (AFP) levels, and radiological characteristics for stratification of recurrence risk (8–10). However, these MVI-centric nomograms fail to address heterogeneous outcomes in MVI-negative cohorts, which continue to exhibit a clinically significant recurrence rate (41.8%) (11). This prognostic ambiguity leaves clinicians without robust tools to identify high-risk MVI-negative subgroups that require intensified follow-up or preventive interventions.

To bridge this translational gap, we developed a novel nomogram specifically tailored to patients with MVI-negative HCC undergoing curative resection. Our model integrates preoperative tumor biological signatures, dynamic serum biomarker profiles, host systemic inflammatory indices, and postoperative immunohistochemical markers to characterize occult biological aggressiveness beyond conventional histopathological criteria. In the current work, we developed a novel nomogram to identify MVI-negative HCC patients at high risk of recurrence after curative resection, thereby providing an individualized tool for prognostic prediction and postoperative surveillance in this specific subpopulation.

## Materials and methods

### Sample size calculation

The sample size estimation for developing the multivariable prognostic model with binary outcomes was performed in accordance with methodological recommendations described by Riley et al (12, 13). Using the “pmsampsize” package in R (R Core Team; R Foundation for Statistical Computing, Vienna, Austria), key parameters were predefined, including a target C-statistic of 0.75, 5 candidate predictors, and an anticipated event prevalence of 60%. The analysis yielded a minimum sample size of 369 participants, corresponding to 222 observed events, to ensure adequate statistical power. This calculation resulted in an event-per-predictor ratio of 44.28, which substantially exceeded the recommended threshold of 10 events per-predictor variable to maintain model stability and minimize overfitting risks in prognostic modeling studies.

### Study population

This study consecutively screened 547 treatment-naïve patients with HCC who underwent curative resection between January 2016 and March 2022 at 2 tertiary centers: the Eastern Hepatobiliary Surgery Hospital (Shanghai; training cohort, n = 375); and the Affiliated Suqian Hospital of Xuzhou Medical University (Suqian City, China; external validation cohort, n = 172). The final analytical sample of 375 patients with 247 outcome events in the training cohort exceeded the minimum required sample size of 369, as calculated through *a priori* power analysis.

The inclusion criteria were as follows: pathologically confirmed primary HCC; MVI-negative status; Barcelona Clinic Liver Cancer (BCLC) stage 0-B; treatment-naïve status before surgery; and complete clinicopathological records and follow-up data. The exclusion criteria were as follows: non-HCC malignancies; preoperative chemotherapy, targeted therapy, immunotherapy, radiofrequency ablation, transarterial chemoembolization, or radiotherapy; MVI-positive status; postoperative adjuvant therapy recipients; and incomplete follow-up.

The external validation cohort maintained identical inclusion/exclusion protocols, except for geographical independence, with all surgeries performed by different surgical teams using comparable techniques.

### Data collection

**Figure 1:**
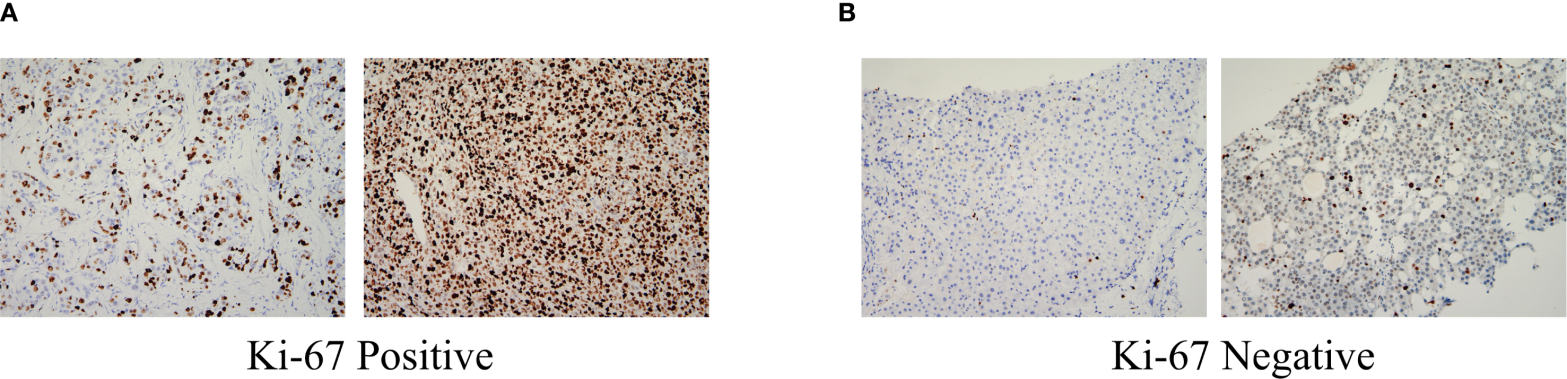
Pathologically, HCC (Immunohistochemistry, ×200): **(A)** Ki-67 Positive:nuclear staining in ≥ 20% of tumor cells; **(B)** Ki-67 Negative:nuclear staining in < 20% of tumor cells. HCC, Hepatocellular carcinoma.

The Institutional Ethics Committee approved the study protocol and written informed consent was obtained from all participants before data collection. Comprehensive baseline characteristics of patients undergoing curative resection were systematically collected from electronic medical records and standardized clinical databases. The data encompassed the following: demographics (age and sex were recorded to evaluate population characteristics); clinical history (pre-existing conditions, such as hypertension, diabetes mellitus, and previous antiviral therapy) was documented to assess comorbidities and treatment history; radiological and pathological features (liver cirrhosis status, Child–Pugh grade, BCLC stage, tumor number, and maximal tumor diameter) were evaluated using preoperative imaging (contrast-enhanced computed tomography [CT]/magnetic resonance imaging [MRI]) and histopathological reports; laboratory parameters (hematological and biochemical profiles were obtained within 1 week before ablation, including white blood cell (WBC) count, neutrophil count, lymphocyte count, platelet count, hemoglobin, alanine aminotransferase, aspartate aminotransferase, total bilirubin, albumin (ALB), γ-glutamyl transpeptidase, alkaline phosphatase, pre-ALB (PALB), AFP, and AFP-L3 levels; postoperative pathological indicators (the proliferation index Ki-67 was analyzed in the resected tumor specimens to assess cellular proliferation activity. Ki-67 positivity was defined as nuclear staining in ≥ 20% of tumor cells, with values < 20% categorized as negative (14), typical Ki-67 staining images show in **Figure 1**). All data were independently verified by 2 researchers to ensure accuracy and consistency. Missing values were addressed using predefined protocols, including exclusion or imputation, based on clinical plausibility.

In addition, 4 composite indices were calculated: neutrophil-to-lymphocyte ratio (NLR; neutrophil count/lymphocyte count); platelet-to-lymphocyte ratio (PLR; platelet count/lymphocyte count); systemic immune-inflammation index (SII; neutrophil count × platelet count/lymphocyte count); and Prognostic Nutritional Index (PNI; albumin [g/L] + 5 × lymphocyte count [×10^9^/L]) (15). The optimal cut-off values for the NLR, PLR, SII, and PNI were determined by maximizing the Youden index, and patients were stratified into high- and low-risk groups based on these thresholds.

### Postoperative follow-up

After discharge, serum AFP and AFP-L3 tests, abdominal ultrasonography, contrast-enhanced CT, MRI, and chest radiography were performed monthly for the initial 3 months, and once every 3 months thereafter. For patients without HCC recurrence 2 years postoperatively, recurrence monitoring was performed every 6 months. Cancer recurrence was defined as the emergence of new intrahepatic or extrahepatic cancer foci with typical imaging features consistent with HCC on enhanced CT or MRI, with or without elevated serum AFP and AFP-L3 levels (**Supplementary Figure S1**). When there was clinical suspicion of postoperative recurrence of HCC or distant metastases, CT, whole-body bone scan, or angiography was performed. Recurrence-free survival (RFS) was calculated from the date of initial hepatectomy to the date of recurrence or last follow-up. Data were censored up to March 31, 2024.

### Statistical analysis

Continuous variables are expressed as mean ± standard deviation and categorical variables as count with percentage, with intergroup comparisons performed using Student’s *t*-test for continuous variables and the chi-squared test for categorical variables. All statistical tests were two-sided and differences with *p* < 0.05 were considered to be significant. All analyses were performed using R version 4.2.2. To identify postoperative predictors of RFS in patients with HCC, a dual analytical approach was used comprising a random survival forest (RSF) model configured with 1000 trees and node size optimization via out-of-bag error minimization to manage high-dimensional feature interactions and multivariable Cox regression adjusted for competing risks, with proportional hazards assumptions verified through Schoenfeld residual analysis. Significant predictors from both models were integrated into a clinically oriented nomogram for individualized probability estimation of RFS. Model discrimination was assessed using Harrell’s concordance index (C-index), time-dependent receiver operating characteristic (ROC) curve analysis at 1-, 3-, and 5-year intervals, calibration plots with Brier scores, and decision curve analysis (DCA) for clinical utility quantification. External validation was conducted using a temporally independent cohort from the Affiliated Suqian Hospital of Xuzhou Medical University. Using the optimal cut-off value for nomogram scores, patients were stratified into low- and high-risk subgroups, with survival disparities validated through Kaplan–Meier analysis (log-rank test) and cumulative recurrence rate comparisons at 1-, 3-, and 5-year intervals.

## Results

### Clinicopathological characteristics of the training and validation cohorts

The study cohort comprised 547 patients with MVI-negative HCC who underwent curative hepatectomy between January 2016 and March 2022, including 375 from the Eastern Hepatobiliary Surgery Hospital (i.e., training cohort) and 172 from the Affiliated Suqian Hospital of Xuzhou Medical University (i.e, external validation cohort). A comprehensive comparison of baseline characteristics demonstrated comparable demographic and clinical profiles between the cohorts (**Table 1**). The mean age of the training cohort was 53.01 years versus (vs.) 51.39 years for the validation cohort (*p* = 0.086), with a comparable proportion of males (73.86% vs. 71.51%). Critical tumor characteristics, including BCLC staging distribution, median tumor diameter, and tumor number, exhibited no significant intergroup differences. Laboratory parameters and postoperative recurrence rates further confirmed cohort homogeneity. These rigorously matched baseline features ensured the methodological validity of subsequent prognostic modeling analyses.

**Table 1:** Comparison of clinical data between Training cohort and External validation cohort.

Variables	Total	Training cohort	External validation cohort	P value
	N=547	N=375	N=172	
Gender (Male/Female)	400/147	277/98	123/49	0.564
Age (years old)	52.50 ± 10.26	53.01 ± 10.35	51.39 ± 9.97	0.086
HBV-DNA (IU/ml) (< 1000/≥1000)	251/296	180/195	71/101	0.143
Cirrhosis (yes/no)	446/101	300/75	146/26	0.172
Tumor number (single/multiple)	456/91	319/56	137/35	0.114
Tumor size (≤50mm/>50mm)	352/195	242/133	110/62	0.895
Capsule (yes/no)	270/277	177/198	79/93	0.136
BCLC (0-A/B)	506/41	350/25	156/16	0.277
RM (<10mm/≥1mm)	114/433	85/290	29/143	0.121
TB (mmol/L)	15.11 ± 6.91	15.37 ± 7.08	14.61 ± 6.52	0.250
ALT (U/L)	33.59 ± 16.28	33.13 ± 16.50	34.60 ± 15.80	0.328
AST (U/L)	39.52 ± 70.43	40.35 ± 76.58	37.71 ± 54.86	0.683
ALB (g/L)	42.16 ± 4.37	42.16 ± 4.45	42.15 ± 4.22	0.987
PALB (g/L)	206.19 ± 65.79	206.69 ± 64.94	205.09 ± 67.64	0.792
GGT (U/L)	58.34 ± 62.49	57.63 ± 61.95	59.88 ± 63.81	0.696
AKP (U/L)	83.66 ± 33.25	84.25 ± 34.36	82.37 ± 30.75	0.540
WBC (×10^9/L)	5.03 ± 1.30	5.01 ± 1.24	5.08 ± 1.40	0.555
AFP (<400/≥400ng/mL)	339/208	242/133	97/75	0.069
AFP-L3 (positive/negative)	177/370	114/261	63/109	0.148
NLR (high/low)	357/190	237/138	120/52	0.134
PLR (high/low)	348/199	237/138	111/61	0.763
SII (high/low)	370/117	258/117	112/60	0.393
PNI (high/low)	446/101	311/64	135/37	0.214
Ki-67 (positive/negative)	262/285	173/202	89/83	0.223
Recurrence	361	247	114	0.925

RM, resection margin; TB, total bilirubin; AFP, alpha-fetoprotein; ALT, alanine aminotransferase; AST, aspartate aminotransferase; ALB, albumin; PALB, pre-albumin; GGT, gamma-glutamyl transpeptidase; AKP, Alkaline phosphatase; WBC, White blood cell; SII, Systemic Immunity Inflammation Index; PLR, Platelet-to-lymphocyte ratio; NLR, Neutrophil-to-Lymphocyte Ratio; PNI, Prognostic Nutritional Index.

### Identifying risk factors for RFS in the training cohort

**Figure 2:**
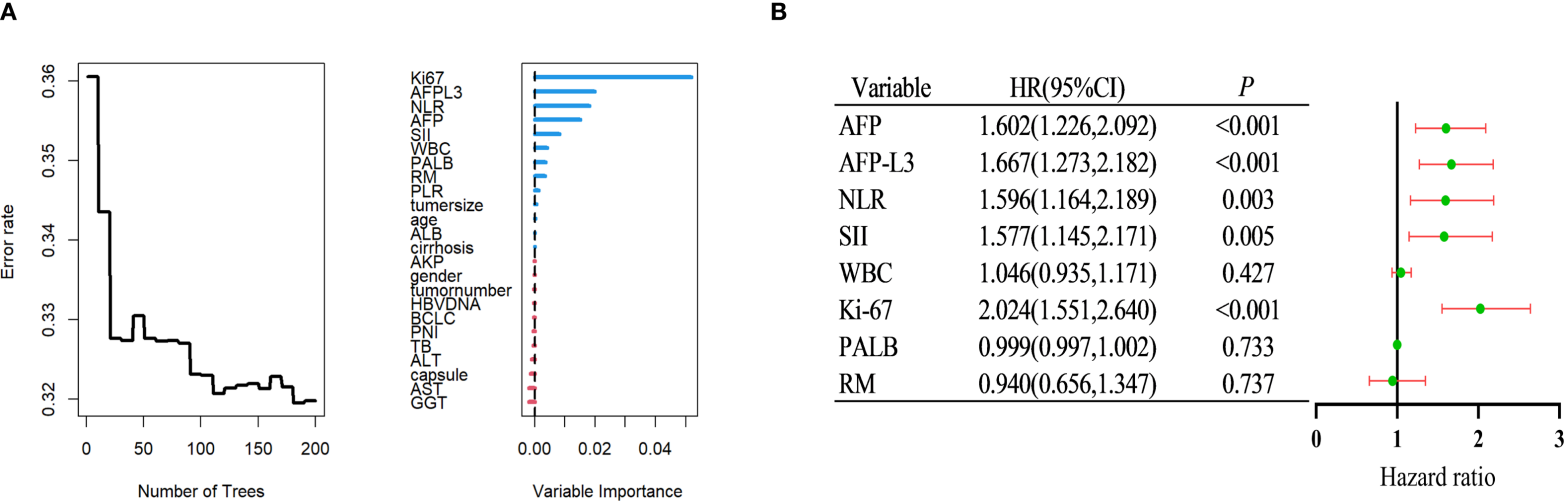
Identify independent risk factors for postoperative recurrence of HCC within the training group. **(A)** Using random survival forest to screen factors. **(B)** Multivariate Cox regression analysis. HCC, Hepatocellular carcinoma.

This section comprehensively reports the potential risk factors influencing RFS in patients with HCC after surgery by integrating RSF with multivariate Cox regression analysis. Initially, an RSF algorithm was used for preliminary variable selection. Using this algorithm, a set of variables with a significant impact on RFS was successfully identified. The top 8 key variables selected by the RSF algorithm and ranked according to importance are listed in **Figure 2A**. These included Ki-67, AFP-L3, NLR, AFP, SII, WBC count, PALB level, and resection margin (RM).

Subsequently, a more rigorous screening process was performed using multivariate Cox regression analysis, which is a statistical technique frequently used to assess the impact of multiple variables on survival. By accounting for potential confounding factors, it was possible to accurately measure the influence of each variable, thereby identifying independent risk factors. As depicted in **Figure 2B**, it was confirmed that Ki-67, AFP-L3, NLR, AFP, and SII were independent risk factors for RFS (multivariate Cox analysis).

### Construction of an RFS prognostic nomogram in the training cohort

**Figure 3:**
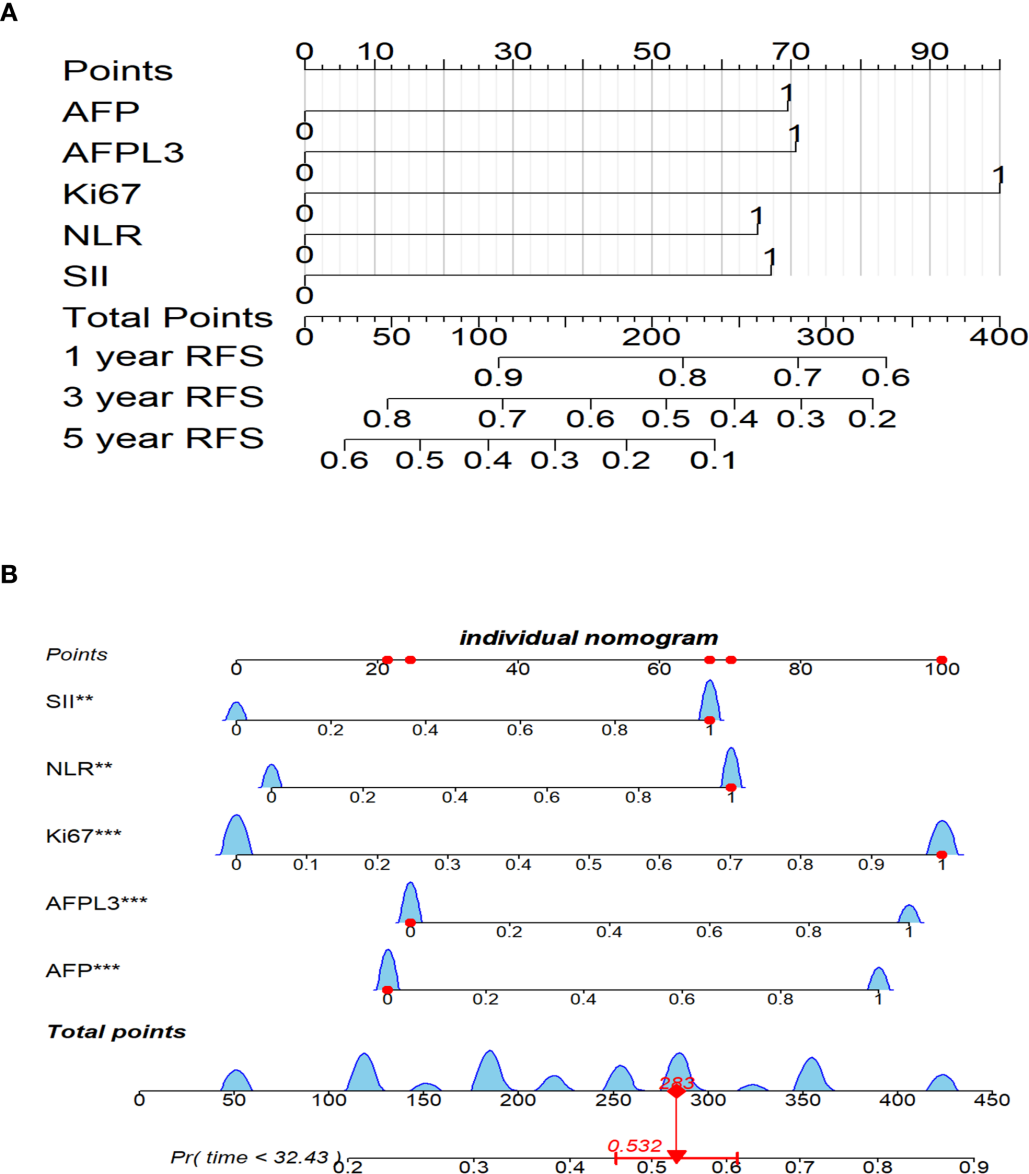
Nomogram for postoperative RFS prediction in HCC patients. **(A)** Nomogram for 1-, 3-, and 5-year postoperative RFS. **(B)** Nomogram for a specific patient. **P<0.01, ***P<0.001. HCC, Hepatocellular carcinoma; RFS, recurrence-free survival.

Using multivariate Cox regression analysis, it was determined that Ki-67, AFP-L3, NLR, AFP, and SII were independent prognostic factors for RFS. To effectively depict the influence of these variables on RFS, a nomogram was developed, as illustrated in **Figure 3A**. In this nomogram, each variable is allocated a specific number of points reflecting its significance and presence. The total accumulated points were then used to calculate a comprehensive score that was aligned with a predetermined outcome scale to determine the likelihood of a specific clinical event. For example, a patient exhibiting positive Ki-67, elevated SII, elevated NLR, an AFP level < 400 ng/mL, and a negative AFP-L3 status accumulated an approximate total score of 282, translating to a 53.2% chance of experiencing postoperative recurrence (**Figure 3B**). Consequently, the nomogram provides a more practical approach for clinical use than traditional mathematical formulae.

**Figure 4:**
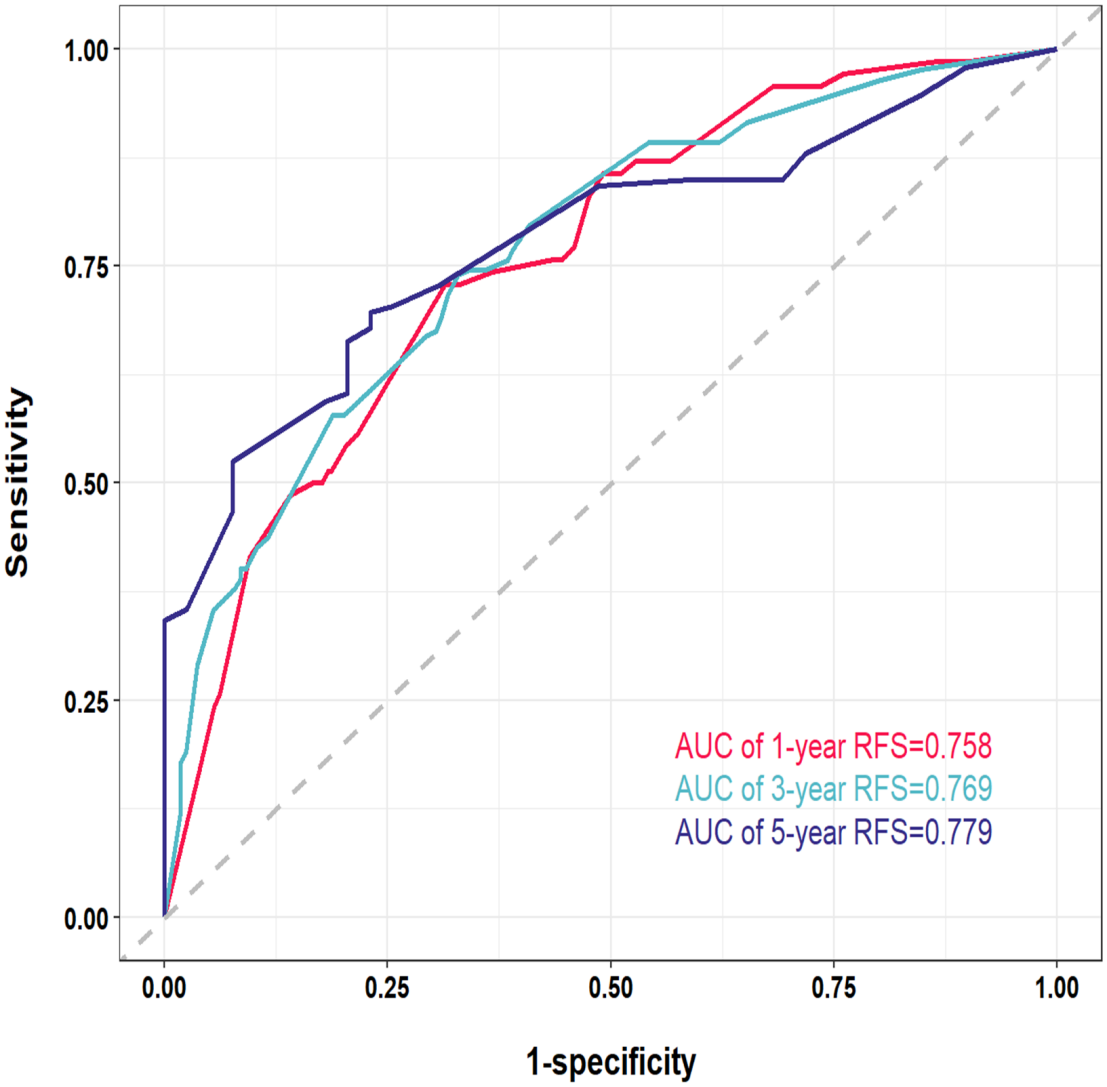
Receiver operating characteristic (ROC) curves of the nomogram for 1-, 3-, and 5-year in the training cohort. AUC, area under the curve.

The nomogram prediction model is designed to provide precise estimates of the likelihood of events occurring within a specified timeframe. Consequently, the nomograms were assessed using discrimination, calibration, and clinical applicability metrics. Discrimination evaluates the capacity of the model to differentiate between patients who experience an event and those who do not, and is quantified through 2 key metrics: the C-index; and the receiver operating characteristic (ROC) curve. In the training cohort, the C-index for the developed nomogram model exceeded 0.7, as illustrated in **Supplementary Figure S2**, signifying that the model proficiently distinguished between instances of recurrence and non-recurrence following surgical intervention for liver cancer. Additionally, ROC curves were charted for 1-, 3-, and 5-year RFS within the training cohort (**Figure 4**). Findings revealed that the area under the ROC curve (AUC) values for forecasting 1-, 3-, and 5-year RFS were 0.758, 0.769, and 0.779, respectively, indicating that the model demonstrated robust performance in predicting RFS.

**Figure 5:**
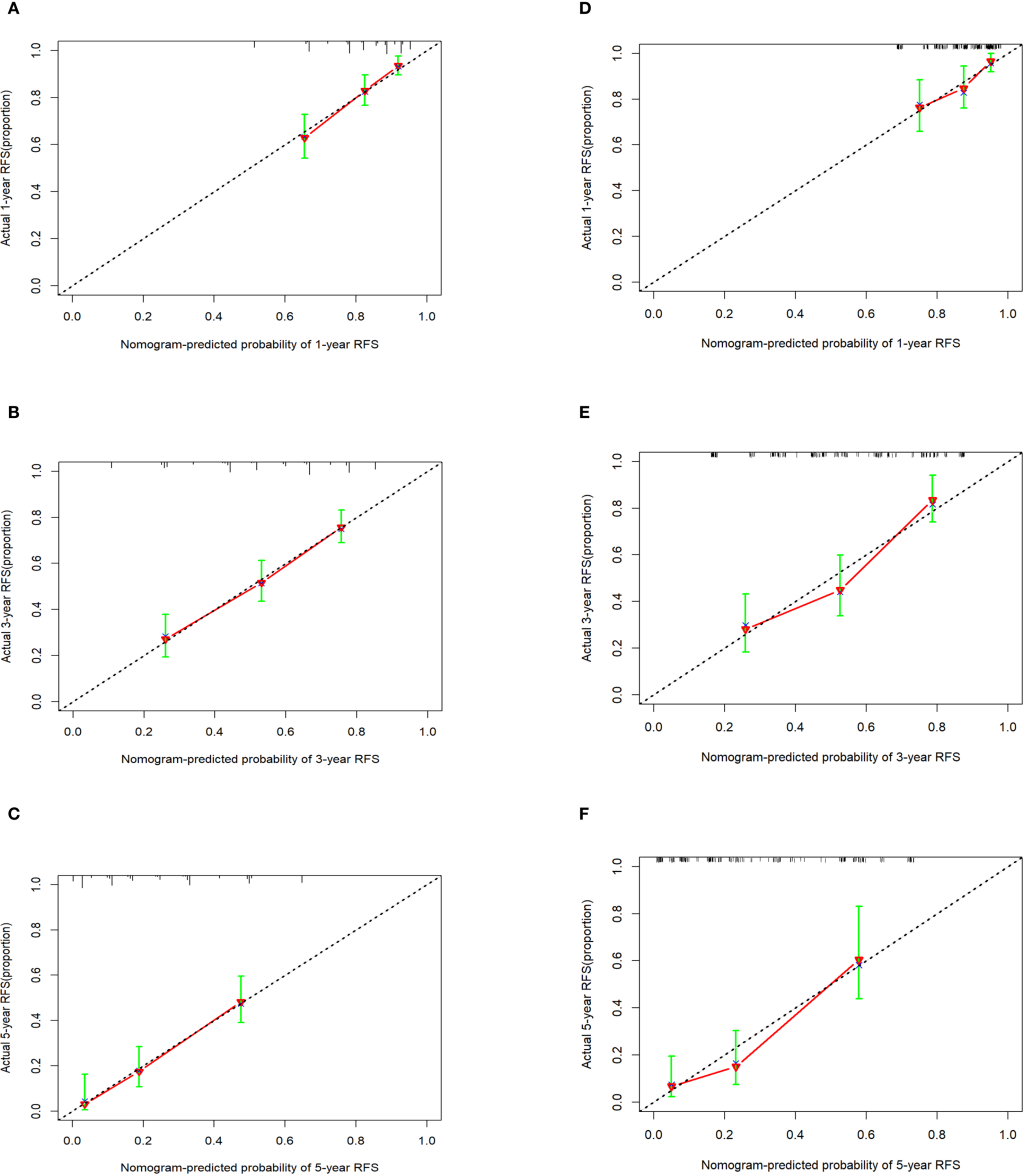
Calibration curves of the nomogram in the training cohort and the external validation cohort. **(A-C)** 1-, 3-, and 5-year calibration curve in the training cohort. **(D-F)** 1-, 3-, and 5-year calibration curve in the external validation cohort.

Calibration curves were used to evaluate the extent of divergence between the anticipated and actual outcomes generated by the model. The proximity of the solid line (which reflects the predicted probabilities according to the model) to the dashed line (representing the actual observed probabilities) within the calibration curves signified a strong alignment between the model’s predicted probabilities for the 1-, 3-, and 5-year RFS and the actual observed probabilities in the patient cohort (**Figures 5A–C**).

**Figure 6:**
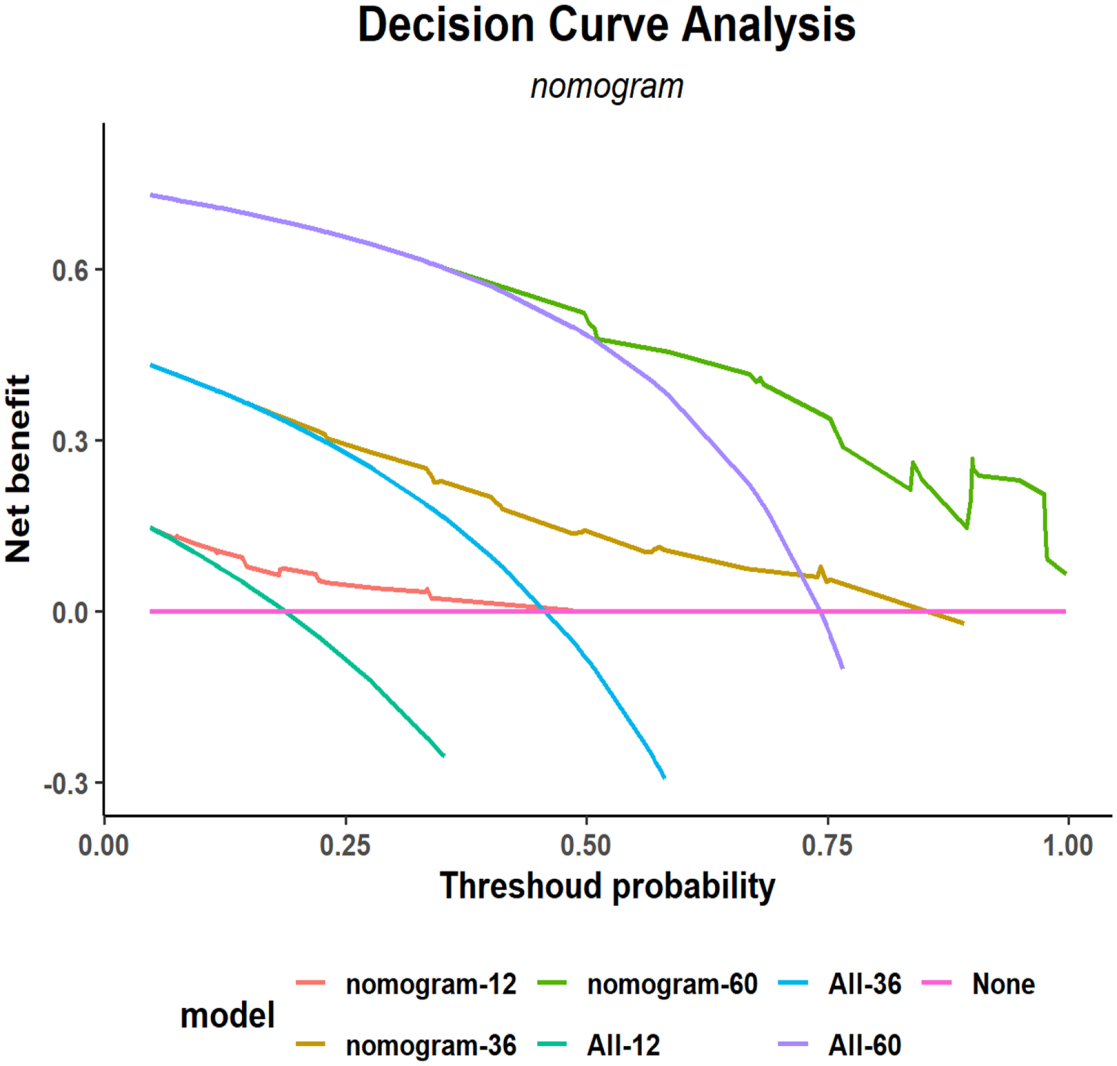
Decision curve analysis (DCA) of the nomogram for 1-, 3-, and 5-year postoperative RFS in the training cohort. RFS, recurrence-free survival.

A decision curve analysis (DCA) graph was used to assess the clinical utility of the prediction model across varying threshold probabilities. In this DCA representation, the x-axis denotes the risk threshold, and the y-axis illustrates the net benefit. A DCA curve that significantly exceeds the extreme curve suggests that the prediction model has enhanced clinical applicability. As depicted in **Figure 6**, DCA indicated that the nomogram model yielded substantial net benefits for 1-, 3-, and 5-year RFS within the training cohort.

### External validation of the RFS prognostic nomogram

External validation of the nomogram using an independent dataset is crucial step for clinical generalization. In this study, the external dataset comprised 172 patients. The validation cohort demonstrated that the C-index of the nomogram was > 0.7 (**Supplementary Figure S3**), with an AUC for 1-year, 3-year, and 5-year RFS rates of 0.7017, 0.735, and 0.756, respectively (**Supplementary Figure S4**). Calibration plots confirmed good agreement between the predicted and observed RFS probabilities (**Figures 5D–F**). Furthermore, DCA indicated that the nomogram in the validation cohort had good net benefits for 1-, 3-, and 5-year RFS (**Supplementary Figure S5**).

### Subgroup analysis of the prognostic nomogram

To further evaluate the performance of the nomogram, subgroup analyses based on the selected variables were performed and compared the results with those of the overall cohort (**Table 2**). The model demonstrated a better discriminative ability in the validation set than that in the training set (C-index, 0.712 vs. 0.703), indicating good generalizability without overfitting. Subgroup analysis revealed consistent performance in patients with BCLC stage 0–A and across tumor size subgroups (≤ 5 cm vs. > 5 cm), further supporting the robustness of the model.

**Table 2:** Results of the subgroup analysis of the prognostic nomogram.

Dataset	Subgroup	No.of patients	C.index	AUC.1year	AUC.3years	AUC.5years
Training	Overall	375	0.703 (0.659-0.726)	0.758	0.769	0.779
	BCLC 0-A	350	0.688 (0.653-0.722)	0.746	0.769	0.767
	BCLC B	25	0.746 (0.629-0.862)	0.861	0.780	0.935
	Tumor ≤5 cm	242	0.699 (0.655-0.743)	0.755	0.776	0.757
	Tumor >5 cm	133	0.690 (0.640-0.740)	0.770	0.757	0.799
Validation	Overall	172	0.712 (0.665-0.759)	0.717	0.735	0.756
	BCLC 0-A	156	0.696 (0.647-0.746)	0.710	0.725	0.751
	BCLC B	16	0.908 (0.811-1.000)	0.846	0.952	1.000
	Tumor ≤5 cm	110	0.697 (0.637-0.757)	0.699	0.709	0.818
	Tumor >5 cm	62	0.748 (0.671-0.824)	0.762	0.799	0.717

### Risk stratification of RFS prognostic nomogram

**Figure 7:**
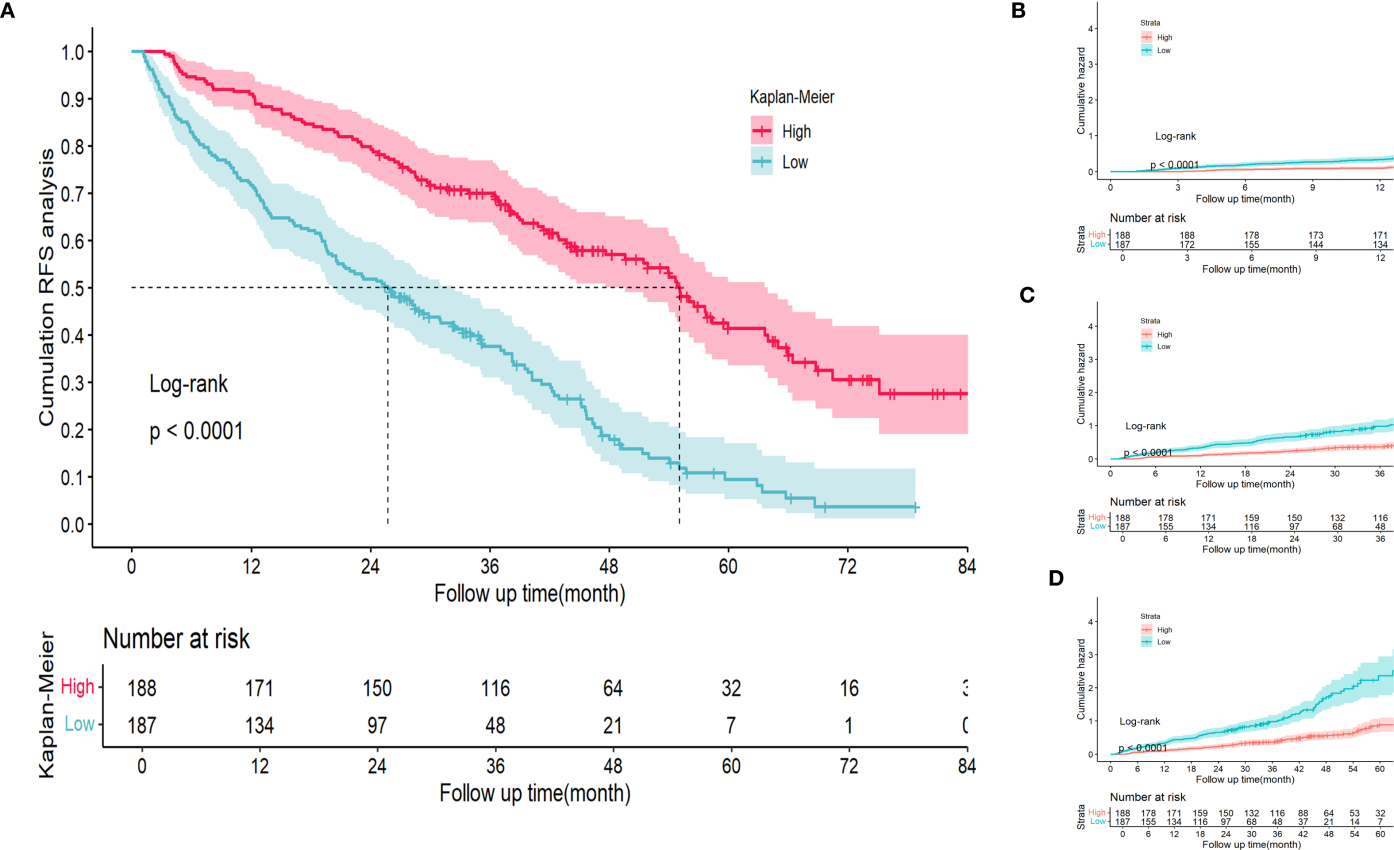
Kaplan Meier curve analysis of different risk groups stratified by nomogram scores in the training cohort. **(A)** RFS Kaplan Meier curve. **(B-D)** 1-, 3-, and 5-year time points cumulative RFS Kaplan Meier curve. RFS, recurrence-free survival.

Within the training cohort, patients were stratified into 2 distinct risk groups—low and high—based on the optimal cut-off values derived from the nomogram points. This stratification aimed to evaluate the effectiveness of the nomogram in accurately distinguishing populations with different recurrence risk profiles. Kaplan–Meier curves were used to compare the RFS between the low- and high-risk groups. In the training cohort, a significant difference in recurrence risk was observed between the low- and high-risk groups (*p* < 0.05) (**Figure 7A**). This indicated that patients in the low-risk group had a better prognosis, whereas those in the high-risk group exhibited a higher propensity for recurrence. Additionally, cumulative recurrence rate survival curves were plotted for the 1-, 3-, and five-year time points (**Figures 7B–D**), respectively, based on the aforementioned stratification, and demonstrated that the nomogram effectively discriminated between populations with different recurrence risk characteristics at various time points.

## Discussion

HCC is a major global health concern, accounting for approximately 75%–85% of all primary liver cancers, and ranks as the sixth-leading cause of cancer worldwide and the third-leading cause of cancer-related mortality (1). Despite advances in treatment options, including surgical resection and liver transplantation, the prognosis of HCC remains poor, with high recurrence rates following curative treatment, necessitating improved prognostic tools and management strategies (16). MVI has been widely validated as a critical independent predictor of postoperative recurrence in HCC, and numerous recurrence risk stratification models (e.g., nomograms and artificial intelligence algorithms) have been developed by incorporating MVI status as a cornerstone variable (10, 17, 18). However, emerging evidence suggests that recurrence patterns in MVI-negative patients differ fundamentally from those in patients with MVI-positive HCC. Specifically, MVI-positive tumors are predominantly associated with early intrahepatic metastases due to vascular dissemination, whereas late recurrences in those with MVI-negative disease may originate from *de novo* carcinogenesis driven by persistent underlying liver pathologies such as metabolic dysfunction or chronic inflammation (19).

In this study, we developed and validated a novel nomogram for predicting RFS in patients with MVI-negative HCC after curative hepatectomy. By integrating RSFs with multivariate Cox regression analysis, we identified 5 independent risk factors for RFS: Ki-67, AFP-L3, NLR, AFP, and SII. The nomogram demonstrated good discrimination, calibration, and clinical utility in both the training and external validation cohorts, providing a valuable tool for personalized risk stratification and clinical decision-making in this specific patient population.

The independent risk factors identified in this study have previously been associated with HCC prognosis (20–23), yet their combined impact on RFS after curative resection in patients with MVI-negative disease has not been fully elucidated. The exclusion of MVI from our analysis enabled us to focus on other factors that may influence recurrence in this subset of patients. Ki-67, a nuclear protein expressed in proliferating cells, is a strong predictor of RFS, even in the absence of MVI (24). This suggests that tumor cell proliferation plays a crucial role in recurrence, irrespective of MVI status. Ki-67 is a significant prognostic biomarker in a range of cancers, particularly HCC. The increased expression of Ki-67 is correlated with heightened immune infiltration of diverse immune cell populations, notably functional T cells, as well as CD4-positive (+) and CD8+ T cell subsets. Furthermore, Ki-67 is strongly associated with T cell exhaustion and plays an essential role in facilitating T cell dysfunction in HCC (25). According to the study by Gao et al. (26), patients who did not exhibit a reduction in the Ki67 index after chemotherapy had a significantly lower median progression-free survival than those in whom the Ki67 index decreased (8.5 months vs. 20 months; hazard ratio [HR] 4.834; *p* = 0.012). Additionally, M1 macrophages were significantly less abundant in the group with a reduced Ki67 index than that in the group with an increased Ki67 index (*p* < 0.001). This suggests that a lower Ki67 index enhances the immune response to chemotherapy, whereas a higher index is associated with reduced efficacy. This suggests that Ki-67 may promote tumor progression by altering the tumor immune microenvironment, leading to a poor(er) prognosis. AFP-L3, a subtype of AFP with high specificity for HCC, has also been shown to be correlated with tumor burden and prognosis in patients with HCC (22). The inclusion of AFP-L3 in our nomogram enhanced its ability to predict RFS compared with models relying solely on total AFP.

Inflammatory markers play significant roles in the occurrence, development, and prognosis of liver cancer. Previous studies have shown that NLR, SII, and PLR are associated with poor prognosis in patients with liver cancer. Some nomograms also incorporate inflammatory markers as risk factors in the construction of predictive models (21, 27, 28). In patients MVI-negative HCC, elevated NLR and SII values may indicate a heightened inflammatory state that promotes tumor progression and contributes to poorer outcomes. AFP, a well-established tumor marker for HCC, remained a strong predictor of RFS in our model, which is consistent with previous reports involving patients with MVI-negative disease (11).

Our study had several limitations, the first of which was its retrospective design, which is inherently susceptible to selection bias and may have led to incomplete data collection. Therefore, future validation through prospective studies to confirm our findings with a higher level of evidence is warranted. Second, the nomogram was developed and validated in a Chinese population, and its performance in other ethnic groups requires further investigation. Third, our nomogram did not incorporate other potential prognostic factors, particularly molecular biomarkers. Future studies should aim to integrate such data, possibly by leveraging advanced machine learning algorithms to identify novel robust biomarkers (29), thereby further refining and enhancing the predictive accuracy of this model.

## Conclusion

In conclusion, we developed and validated a novel nomogram for predicting RFS in patients with MVI-negative HCC after curative hepatectomy. Given that our model was constructed exclusively using data from patients with MVI-negative HCC, specific established prognostic factors for overall HCC, such as BCLC stage and tumor size, were not included. Therefore, the present model is specifically intended to predict recurrence risk in patients with MVI-negative HCC. This nomogram, which incorporated 5 independent risk factors, demonstrated good discrimination, calibration, and clinical utility. This is a valuable tool for personalized risk stratification and clinical decision-making in patients with MVI-negative HCC.

## Data Availability

The raw data supporting the conclusions of this article will be made available by the authors, without undue reservation.

## Supplementary material

Supplementary Figure 1 Typical preoperative and postoperative images of tumor recurrence in the training and validation cohorts, with arrows indicating the tumor location.

Supplementary Figure 2 C-index for the developed nomogram in the training cohort.

Supplementary Figure 3 C-index for the developed nomogram in the external validation cohort.

Supplementary Figure 4 Receiver operating characteristic (ROC) curves of the nomogram for 1-, 3-, and 5-year in the external validation cohort. AUC, area under the curve.

Supplementary Figure 5 Decision curve analysis (DCA) of the nomogram for 1-, 3-, and 5-year postoperative RFS in the external validation cohort. RFS, recurrence-free survival.
