# Supplementary figures and images for: Nomogram for predicting postoperative recurrence in patients with microvascular invasion-negative hepatocellular carcinoma: development and validation
Source: Front Immunol. 2025 Oct 7;16:1614392. doi: 10.3389/fimmu.2025.1614392 (PMC12537772; doi:10.3389/fimmu.2025.1614392)

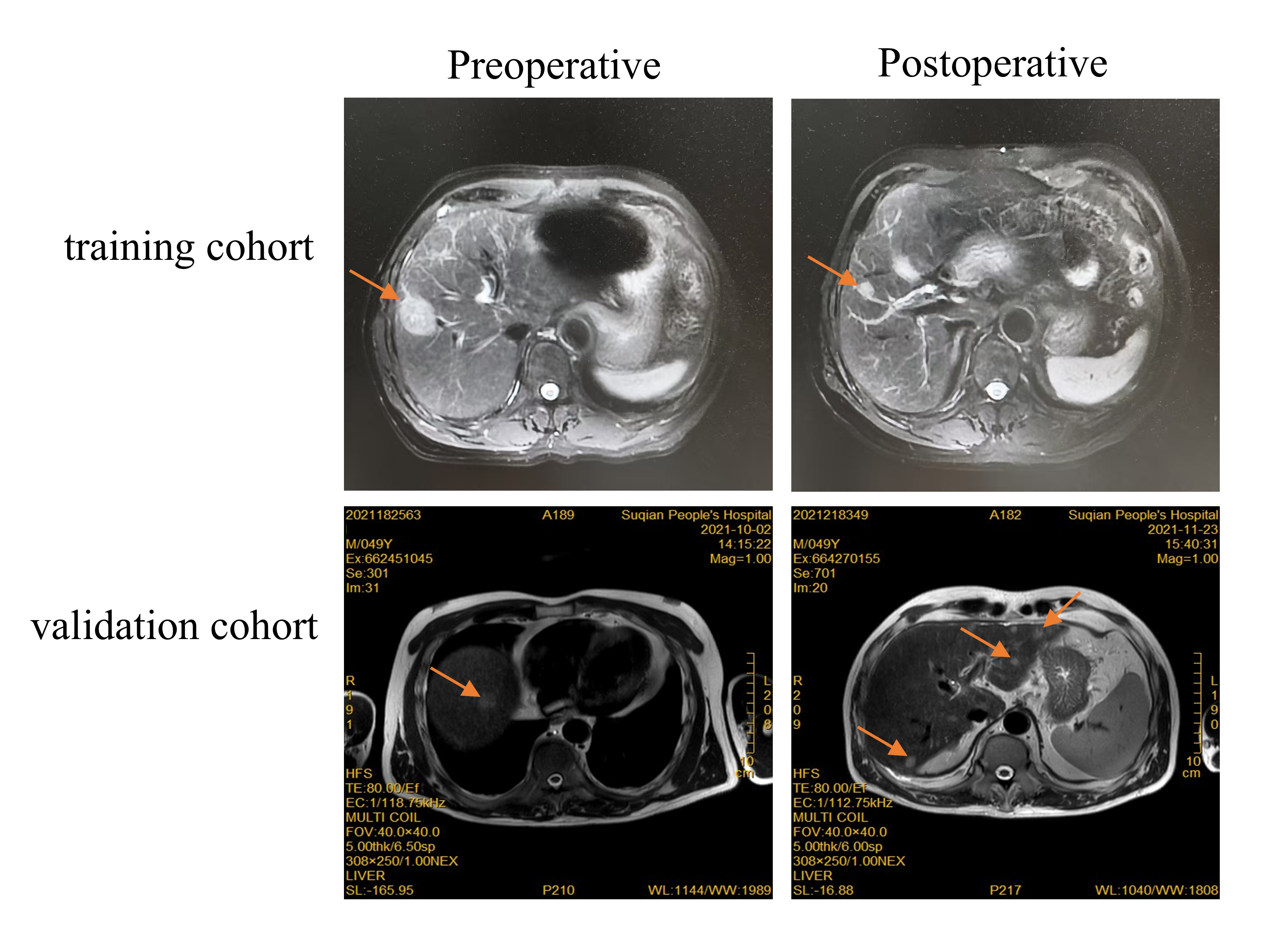

Supplement: Supplementary Figure 1 — Typical preoperative and postoperative images of tumor recurrence in the training and validation cohorts, with arrows indicating the tumor location. [file Image1.tif]

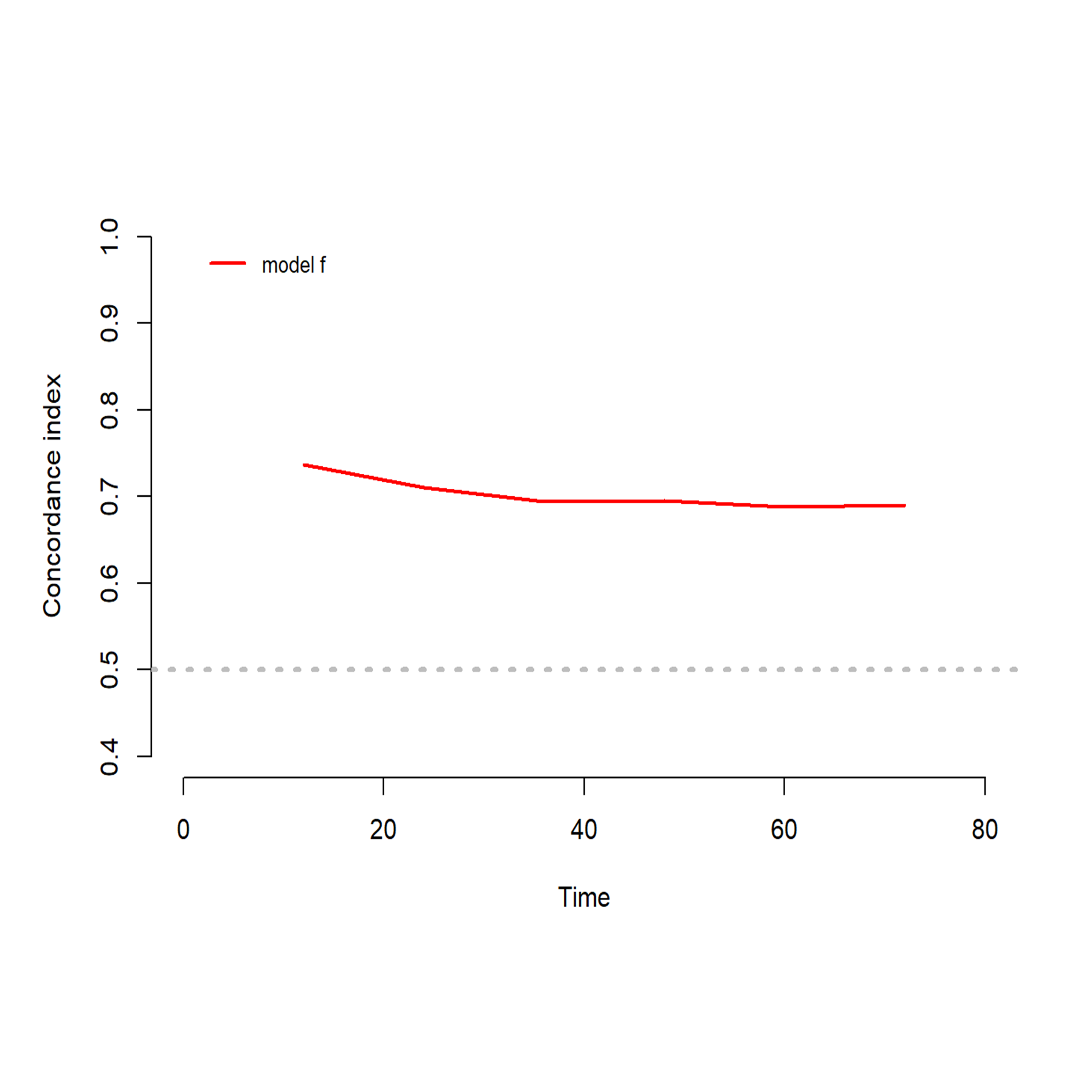

Supplement: Supplementary Figure 2 — C-index for the developed nomogram in the training cohort. [file Image2.tif]

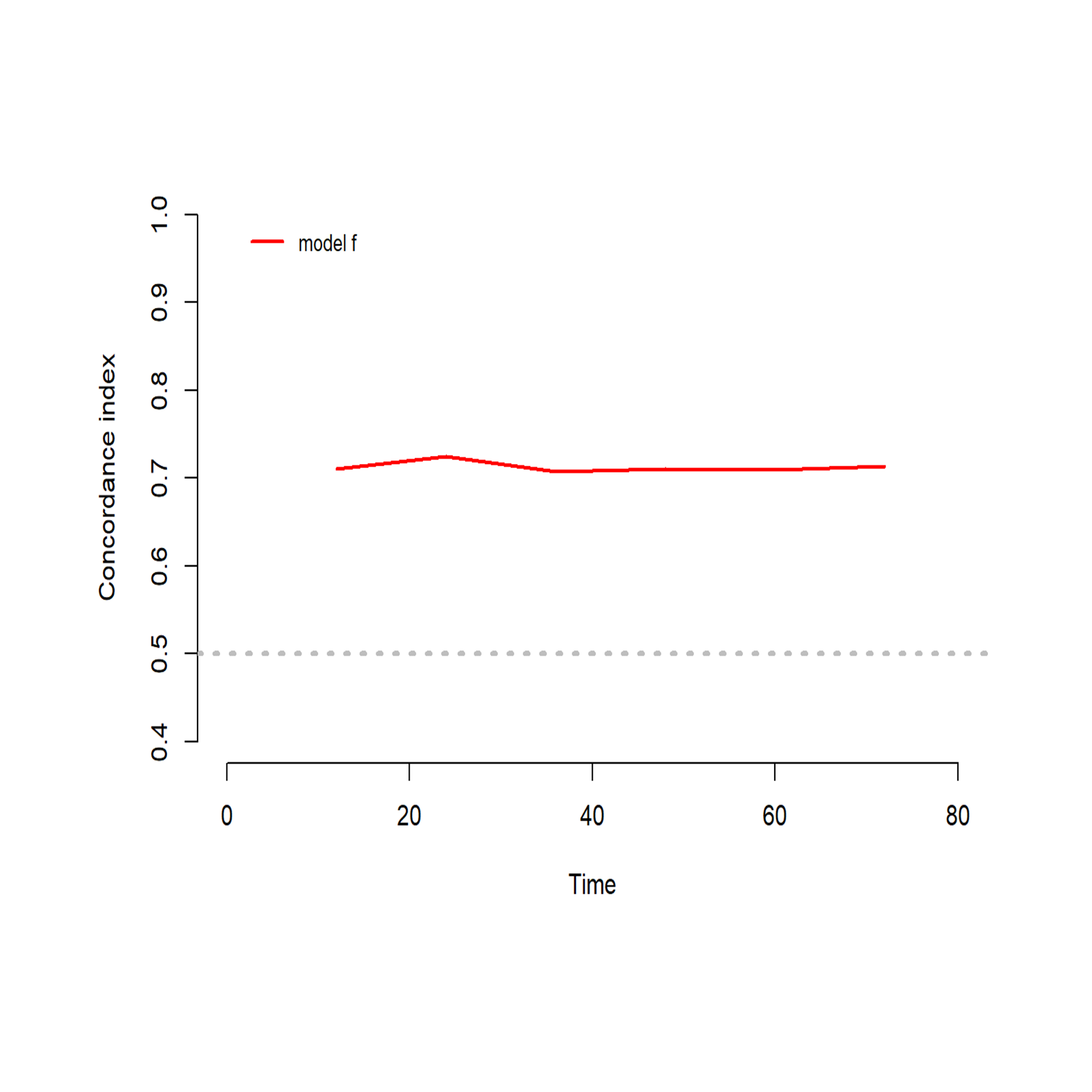

Supplement: Supplementary Figure 3 — C-index for the developed nomogram in the external validation cohort. [file Image3.tif]

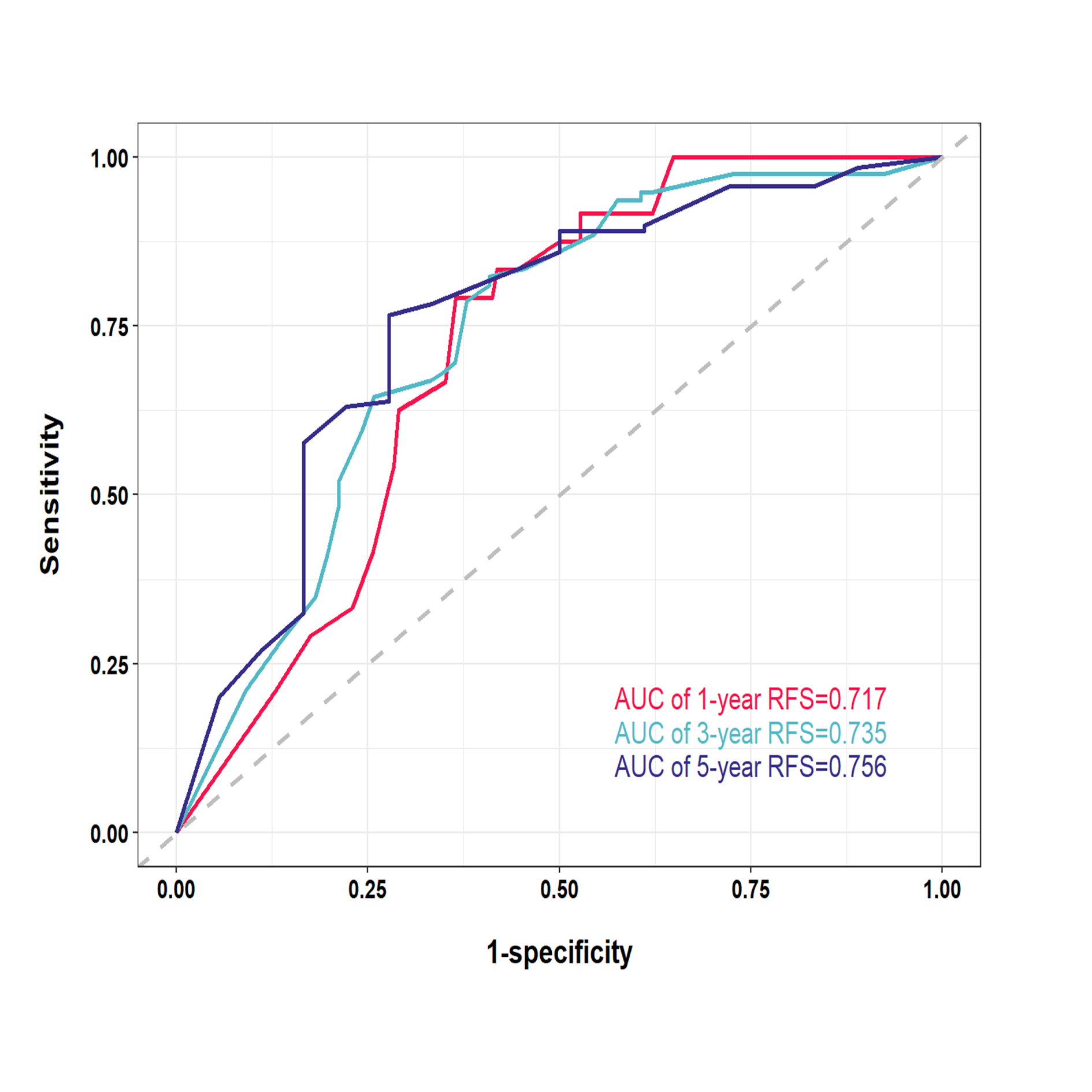

Supplement: Supplementary Figure 4 — Receiver operating characteristic (ROC) curves of the nomogram for 1-, 3-, and 5-year in the external validation cohort. AUC, area under the curve. [file Image4.tif]

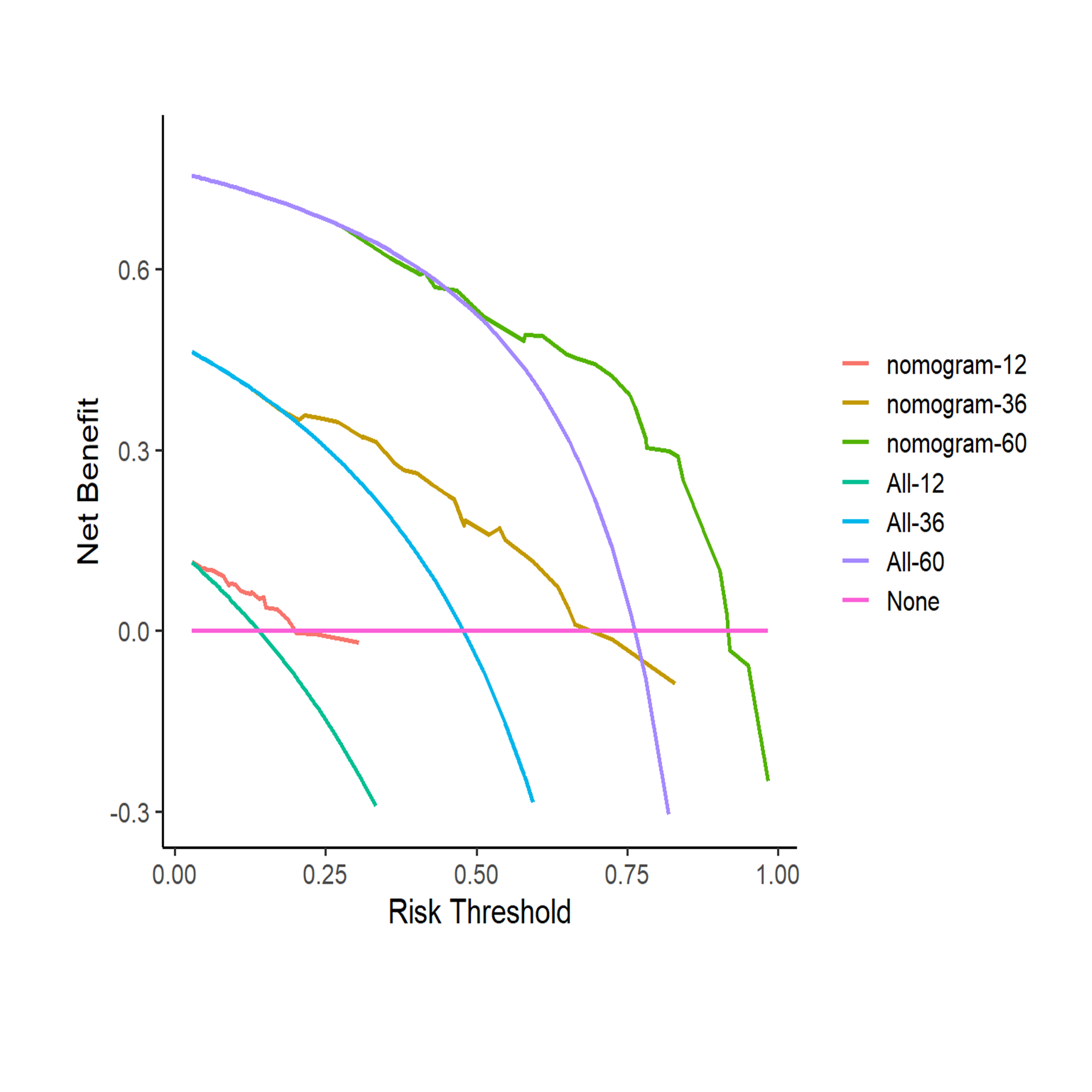

Supplement: Supplementary Figure 5 — Decision curve analysis (DCA) of the nomogram for 1-, 3-, and 5-year postoperative RFS in the external validation cohort. RFS, recurrence-free survival. [file Image5.tif]
